# Depth-dependent flow and pressure characteristics in cortical microvascular networks

**DOI:** 10.1371/journal.pcbi.1005392

**Published:** 2017-02-14

**Authors:** Franca Schmid, Philbert S. Tsai, David Kleinfeld, Patrick Jenny, Bruno Weber

**Affiliations:** 1 Institute of Fluid Dynamics, ETH Zurich, Zurich, Switzerland; 2 Department of Physics, University of California at San Diego, La Jolla, California, United States of America; 3 Section of Neurobiology, University of California, La Jolla, California, United States of America; 4 Institute of Pharmacology and Toxicology, University of Zurich, Zurich, Switzerland; University of Virginia, UNITED STATES

## Abstract

A better knowledge of the flow and pressure distribution in realistic microvascular networks is needed for improving our understanding of neurovascular coupling mechanisms and the related measurement techniques. Here, numerical simulations with discrete tracking of red blood cells (RBCs) are performed in three realistic microvascular networks from the mouse cerebral cortex. Our analysis is based on trajectories of individual RBCs and focuses on layer-specific flow phenomena until a cortical depth of 1 mm. The individual RBC trajectories reveal that in the capillary bed RBCs preferentially move in plane. Hence, the capillary flow field shows laminar patterns and a layer-specific analysis is valid. We demonstrate that for RBCs entering the capillary bed close to the cortical surface (< 400 μm) the largest pressure drop takes place in the capillaries (37%), while for deeper regions arterioles are responsible for 61% of the total pressure drop. Further flow characteristics, such as capillary transit time or RBC velocity, also vary significantly over cortical depth. Comparison of purely topological characteristics with flow-based ones shows that a combined interpretation of topology and flow is indispensable. Our results provide evidence that it is crucial to consider layer-specific differences for all investigations related to the flow and pressure distribution in the cortical vasculature. These findings support the hypothesis that for an efficient oxygen up-regulation at least two regulation mechanisms must be playing hand in hand, namely cerebral blood flow increase and microvascular flow homogenization. However, the contribution of both regulation mechanisms to oxygen up-regulation likely varies over depth.

## Author summary

The brain consumes approximately 20% of the total oxygen used by the human body. An efficient and robust energy supply is essential for the brain’s functioning. The brain is able to up-regulate its oxygen supply in the proximity of neuronal activation. However, the details of the underlying vascular regulation mechanisms remain unknown. To improve the understanding of the blood flow patterns in the cortex we perform numerical simulations in realistic microvascular networks. In contrast to experimental measurements, numerical computations offer the advantage that the whole pressure and flow field is available for analysis. It is well established that the cerebral cortex is organized in laminar fashion and indeed our results reveal that the flow field in the capillary bed shows significant layer-specific differences. Those differences must be taken into account in future numerical and experimental works. Furthermore, it seems likely that multiple regulation mechanisms are playing hand in hand and that their impact differs over depth.

## Introduction

The brain has the ability to locally adapt cerebral blood flow to a locally increased energy demand [1, 2]. Neuronal activity triggers dilation of vessels which leads to an increased blood flow and therewith to an up-regulation of metabolite and oxygen supply. Additionally, further small-scale regulation is likely to take place [3–5]. However, the detailed vascular mechanisms and the signaling pathways between neurons and vessels are still not fully resolved. One of the difficulties in understanding neurovascular coupling lies in the interaction of the different regulation mechanisms playing at approximately the same time but on different spatial scales. The complex topology of the vasculature as well as the high temporal and spatial resolutions required pose further challenges in performing elucidating measurements in large samples.

The most fundamental and most studied regulation mechanism is the increase in blood flow which also is the basis for various measurement techniques such as functional magnetic resonance imaging (fMRI). It is well established that arterioles dilate during neuronal activation and contribute to the up-regulation of blood flow [1, 6]. However, Hall et al. [3] recently observed that capillaries respond to stimulation earlier than arterioles and they speculate that 84% of the blood flow increase results from dilation of capillaries by pericytes. Thus, there is an ongoing debate about which vessel type is mainly responsible for the increase in blood flow.

To answer this question the pressure distribution in cerebral microvascular networks (MVNs) is crucial. Indeed, if pure plasma flow is assumed diameter changes at the location of the largest pressure drop will lead to the largest change in flow rate. Yet, in vivo it is extremely challenging to measure blood pressure in MVNs. Commonly used micropipette measurements are limited to vessels close to the cortical surface and are only applicable to vessels with a diameter > 25 μm [7–9]. Furthermore, many measurements at different locations would be needed to compute the pressure drop in different vessel types, which is not feasible in in vivo studies. Hence, numerical simulations are needed to obtain detailed information on the pressure distribution in MVNs.

On the smaller scale, the effect of neuronal activation on individual capillaries was investigated in multiple studies [4, 5, 10–13] which all agree that red blood cell (RBC) velocity and flux increase in the capillary bed with neuronal activity. Additionally, a homogenization of RBC flux in the capillary bed is observed during activation [4, 5, 13, 14]. Jespersen and Østergaard showed that a reduced capillary transit time heterogeneity (CTH) is beneficial for the overall oxygen extraction fraction (OEF) [15]. Nonetheless, the precise role and impact of the observed phenomena in the up-regulation of oxygen in the brain is still debated.

Another challenge in performing in vivo flow measurements in MVNs is the limited penetration depth of the available measurement techniques. Most in vivo studies in cortical MVNs with resolution on the micrometer scale are limited to a few hundreds of micrometers of the cortex and little is known about the flow patterns deep in the grey matter [3, 4, 10, 12, 13]. This constitutes a major problem, because the neocortex consists of six cortical layers with different neuronal densities and different metabolic demands. Studies on the vasculature showed that vascular densities also vary over cortical depth [16–19] and high-resolution fMRI studies revealed that laminar differences persist in changes in cerebral blood flow (CBF) and cerebral blood volume (CBV) during activation [20, 21]. Furthermore, two studies with measurements of flow properties in individual capillaries down to a depth of 600 μm observed depth-dependent changes in RBC velocity [11, 14]. Hence, laminar differences in flow characteristics seem likely during baseline and activation, but have not been investigated in detail.

We simulate blood flow in three realistic microvascular networks from the mouse parietal cortex [19, 22]. In contrast to previous numerical works in realistic MVNs, which are mostly based on the empirical steady state model derived by Pries et al. [23], we apply a numerical model which directly tracks individual RBCs. Additionally, we present a new approach for assigning appropriate and realistic boundary conditions at the in- and outflows of MVNs.

This work aims to answer questions that are crucial for research on neurovascular coupling and which are extremely difficult to be answered experimentally. We analyze our simulation results with three key questions in mind: (1) Where does the largest pressure drop take place? (2) Are there differences in flow properties over the cortical depth and is it relevant for up-regulation of blood flow? (3) How large is the CTH and what can be concluded from the trajectories of RBCs through the capillary bed?

## Materials and methods

### Microvascular networks

We studied three MVNs from the mouse parietal cerebral cortex. The networks were acquired with the use of two-photon laser scanning microscopy [19, 22, 24]. For details on the data acquisition and the validation of the methods used consult [19, 22, 24] and references therein. To properly differentiate between the different vessel types information on the presence of smooth muscle cells and pericytes would be needed [25]. As this information is not available, Blinder et al. [19] classified the vessels based on their morphology, their diameter and the tracking of penetrating trees into: pial arterioles (PA), descending arterioles and arterioles (DA+A), capillaries (C), venules and ascending venules (V+AV) and pial venules (PV). The vessel classification is extended by a diameter criterion, which requires that two consecutive vessels have a diameter < 7.0 μm until the capillary bed starts. The labeling is further adjusted such that there is no capillary with a diameter > 9.0 μm and no arteriole/venule with a diameter < 6.0 μm. In general MVN 3 has slightly smaller diameters than MVN 1 and 2 and hence, the minimum arteriole/venule diameter had to be set to 4.8 μm in order not to treat most DAs as part of the capillary bed. In the original labeling it is differentiated neither between DAs and As nor between Vs and AVs. Nonetheless, in the course of this work we comment on the impact of introducing this additional classification (S1 Table).

**Fig 1:**
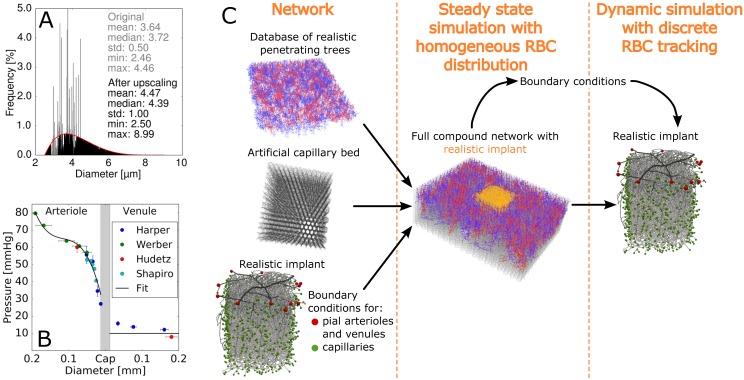
Pre-processing of the microvascular networks and the approach to assign boundary conditions. (A) Histogram-based upscaling approach for microvascular network 1. The grey and the black histogram show the original and the final diameter distribution, respectively. The mean, the standard deviation (std), the maximum value (max) and the minimum value (min) of all capillary diameters are stated in grey and black for the original and the final capillary diameter distribution, respectively. The red curve is the goal beta distribution. (B) Summary of the pressure measurements in the pial vasculature available in literature and the fit we used to assign the pressure boundary conditions at the pial arterioles. At the pial venules we uniformly prescribed a pressure of 10 mmHg. Data from: Harper [7], Werber [8], Hudetz [44], Shapiro [9]. (C) Schematic illustration of the steps of the hierarchical boundary condition approach. On the left the three different components of the full compound network are shown. The red and green spheres in the realistic implant represent the pial and capillary in- and outflows, respectively.

Literature data on capillary diameters in rodents is sparse and varies significantly. Table 1 summarizes some of the available measurements of capillary diameters in rodents. As the mean capillary diameter for all MVNs in this study is < 4.0 μm the vessel diameters are upscaled slightly. We applied a histogram-based upscaling approach based on a beta distribution with a mean of 4.0 μm and a standard deviation of 1.0 μm (Fig 1A). The beta distribution is defined in the diameter range [2.5 μm, 9.0 μm]. Details on the histogram-based upscaling approach are summarized in S1 Fig.

**Table 1:** Overview of literature data on capillary diameters in rodents.

Author	Year	Animal	Capillary diameter	Comment
Craigie [26]	1938	rat	2.9 μm	taken from [27]
Ma et al. [28]	1963	rat	4.2 μm	taken from [27]
Lierse [29]	1974	rat	7.0 μm	taken from [27]
Hudetz et al. [30]	1993	rat	4.4 ± 1.0 μm	-
Kleinfeld et al. [11]	1998	rat	5.0 – 7.0 μm	-
Boero et al. [31]	1999	mouse	3.3 ± 0.4 μm	or smaller depending on brain region
Drew et al. [32]	2011	mouse	2.9 ± 0.5 μm	-
Hall et al. [3]	2014	mouse	4.4 ± 0.1 μm	-
Gutiérrez-Jiménez et al. [14]	2016	mouse	4.0 ± 0.1 μm	-

In Table 2 several characteristic parameters of the MVNs are summarized. All networks are of cuboid shape with a nearly quadratic surface area and a depth such that most grey matter is contained in it.

**Table 2:** Characteristic parameters of the analyzed microvascular networks (MVNs).

MVN	Width	Depth	No. DA	No. AV	No. vessels
1	1.08 mm	1.61 mm	8	30	19 916
2	1.28 mm	1.77 mm	13	40	36 257
3	1.03 mm	1.07 mm	10	21	14 315

DA: descending arteriole, AV: ascending venule.

### Numerical model

The simulation procedure is described in more detail in [33] but briefly summarized below. Our results presented in [33] showed that particularly for the flow in the capillary bed it is crucial to consider the impact of RBCs and hence blood is treated as a biphasic fluid. A short analysis on the impact of RBCs on the flow in realistic microvascular networks is presented in S2 Fig. In the numerical representation the tortuous vessels of the MVN are approximated by straight pipes between two bifurcations. The straight pipe is assigned the same length and resistance as its tortuous equivalent. Therewith, we only neglect the effect of diameter changes along individual vessels.

The flow in the pipe *ij* between the nodes *i* and *j* is computed by Poiseuille’s law

$$q_{ij}=\frac{p_{i}-p_{j}}{R_{ij}^{e}},$$ (1)

where *q*_*ij*_ and $R_{ij}^{e}$ are the flow rate and the effective resistance of pipe *ij*, respectively, *p*_*i*_ and *p*_*j*_ denote the pressure values at the nodes *i* and *j*. The effective resistance $R_{ij}^{e}$ accounts for the presence of RBCs by multiplying the resistance of a circular tube *R*_*ij*_ with the relative apparent viscosity *μ*_*vitro*,*ij*_, which is based on the empirical formulation derived by Pries and Secomb [34]:

$$R_{ij}^{e}=\mu_{vitro,ij}\;R_{ij}=\mu_{vitro,ij}\frac{128\;\mu_{plasma}}{\pi \;d_{ij}^{4}}L_{ij},$$ (2)

where *μ*_*plasma*_ is the plasma viscosity and *d*_*ij*_ and *L*_*ij*_ are the vessel diameter and length, respectively. The relative apparent viscosity *μ*_*vitro*,*ij*_ is a function of the tube hematocrit *ht*_*ij*_ and the diameter of the vessel *d*_*ij*_: *μ*_*vitro*,*ij*_ = *f*(*ht*_*ij*_, *d*_*ij*_). The complete expression and a justification for this choice is given in S1 Text.

RBCs are tracked individually (discrete simulation). According to the Fåhraeus effect the RBC velocity is on average larger than the bulk flow velocity [35]. At divergent capillary bifurcations we assume that the RBCs follow the path of the largest pressure force [36, 37], which is equivalent to the path of the largest bulk flow velocity. Another commonly used bifurcation rule states that the RBC moves into the branch with the largest flow rate [38, 39]. For 91% of all capillary divergent bifurcations in the three MVNs the two bifurcations rules predict the same behaviour (based on the results of pure plasma simulations). Hence, we are confident that the choice of the bifurcation rule, does not greatly affect the overall results. In arteriole divergent bifurcations the RBC motion is more complicated and their separation is described by the phase-separation law introduced by Pries et al. [40]. The RBC tracking is implemented such that an overlapping of RBCs is not possible. For example, at convergent bifurcations RBCs can be blocked temporarily if there is not enough space in the subsequent vessel. As soon as the RBC fits into the next vessel it will proceed.

To reduce the computation time and in contrast to the model introduced in [33] the time step Δ*t* is constant and not a function of the next bifurcation event. Hence, per time step multiple bifurcation events take place. The time step is chosen such that for nearly all vessels (> 99.8%) the following criterion is satisfied:

$$\Delta t\leq \frac{L_{ij}}{v_{ij}},$$ (3)

where Δ*t* is the time step, *L*_*ij*_ and *v*_*ij*_ are the length and the bulk flow velocity of edge *ij*. For each time step two computation steps are performed. In the first one, which is based on the current distribution of RBCs, the pressure and flow field is calculated. Consecutively, the RBCs are propagated and their distribution is updated.

The RBCs are tracked for at least one turn-over time, which is the ratio of the total vascular volume to the sum of all inflows. To minimize the impact of the initial conditions the simulation is run for at least 15 turn-over times before the RBC path is recorded.

### Boundary conditions

A major challenge in simulating blood flow in realistic MVNs is the assignment of appropriate boundary conditions. In Fig 1C an exemplary MVN with all its in- and outflow vertices is illustrated. In most numerical studies only the in- and outflow vertices at the pial level are kept to assign pressure boundary conditions. All in- and outflow branches deeper in the cortex are eliminated or no flow boundary conditions are prescribed [41–43]. The results in [42] show that this approach generally underestimates the flow.

Hence, we introduce a new approach where the realistic MVN is implanted into a large artificial MVN (compound network). In the center of the artificial network a hole of the size of the realistic network is cut. The realistic network is positioned in the cut-out and connected to the artificial network at the deep in- and outflow vertices. By assuming a constant tube hematocrit the pressure distribution for the compound network can be computed (steady state simulation without RBC tracking). The pressure values obtained from the steady simulation are assigned as boundary conditions to the deep in- and outflow vertices of the realistic microvascular network. The simulation with discrete RBC tracking is only executed in the realistic network. Fig 1C illustrates the steps of the hierarchical boundary condition approach.

The artificial network consists of a database of realistic penetrating trees and an artificial capillary bed (Fig 1C). The realistic penetrating vessels have been obtained from the somatosensory cortex of the rat [45]. To account for the differences in size between rat and mouse brain, the arteriole and venule trees have been scaled by the ratio of cortical thicknesses $ct:\;\frac{ct^{mouse}}{ct^{rat}}=0.66$ [46]. The penetrating trees are arranged as a rhombic lattice based on the observations by Blinder et al. [19]. The artificial capillary bed is constructed from a stacked hexagonal network which represents a simplified mesh structure where every bifurcation has three adjacent edges [47, 48]. To finish the artificial MVN the ends of the penetrating trees are connected to the closest capillary vertex.

Literature data on pressure measurements in pial vessels is very sparse [7–9, 44]. We fitted a polynomial of degree 3 to the available pressure measurements in pial arterioles (Fig 1B). The pressure in pial venules depends only weakly on the diameter, hence we uniformly set the pressure to 10 mmHg. The tube hematocrit at all inflow edges is set to the physiological value of 0.3.

In the studied MVNs the deep in- and outflows were already trimmed and hence, in their original configuration it was not possible to implant and connect the realistic MVNs to the artificial one. In order to create in- and outflows over the depth, we cut off 12.5% of the total width on all sides. The average depth of the mouse cortex is 1.2 mm [46]. Hence, we trim the MVNs analyzed at a depth of 1.2 mm to create in- and outflows at the bottom (MVN 3 is trimmed at a depth of 0.95 mm).

### Validation

Simultaneous measurements of flow characteristics in multiple vessels of networks embedded in a tissue volume of > 1 mm^3^ are very challenging to perform in vivo. Recently, Lee et al. [12] presented a promising approach where optical coherence tomography is used to measure the RBC flux in several capillaries at the same time with a temporal resolution of ∼1 s. Other than that, most in vivo measurements only target a very small subset of vessels and average over several seconds.

We validate our simulation setup by comparing our results to two frequently measured flow characteristics: the RBC velocity *v*_*RBC*_ and the RBC flow rate *q*_*RBC*_ (Table 3). In our opinion it is essential to compare flow properties for different vessel types. Furthermore, especially in the DAs a good agreement is crucial, because those vessels are the inflow vessels of the downstream capillary bed. Literature values show that the flow in the microvasculature is heterogeneous and fluctuating in time and hence, the range of measured values is large [49]. All in all, our results are in line with the in vivo measurements and we conclude that the assigned boundary conditions and our numerical framework are appropriate.

**Table 3:** Validation of the simulation results with literature data.

	DA: $q_{RBC}^{in}\;\left[{\text{nl s}}^{-1}\right]$	DA+A: *v*_*RBC*_ [mm s^−1^]	C: *v*_*RBC*_ [mm s^−1^]	C: *q*_*RBC*_ [RBCs s^−1^]
Literature	0.1 − 10.0 [50]	2.0 − 30.0 [50]	mean: 0.4 − 2.0 [4, 10, 11, 13, 14, 51, 52]	mean: 38.6 − 62.0 [13, 14, 53]
MVN 1	0.88 ± 0.87	2.44 ± 4.56	0.82 ± 1.31	59.1 ± 237.6
MVN 2	5.15 ± 8.57	5.28 ± 7.61	1.38 ± 1.96	88.4 ± 574.0
MVN 3	0.96 ± 1.00	2.73 ± 4.97	0.59 ± 0.93	29.8 ± 219.0

$q_{RBC}^{in}$: RBC flow rate in the first segments of the DA, *v*_*RBC*_: RBC velocity in the DA+A, *q*_*RBC*_: RBC flux. The values of the simulation results are given as mean ± standard deviation. For the RBC flux *q*_*RBC*_ the median ± standard deviation are given. DA: descending arteriole, A: arteriole, C: capillary, MVN: microvascular network.

### Data analysis

Our data analysis is based on trajectories of individual RBCs through the MVN. We tracked all RBCs entering the MVN at the pial level. RBCs entering the network at the deep in- and outflows can not be analyzed because the history of the RBC pathway is unknown. Yet, we expect that comparable characteristics along the pathway of those RBCs would be observed.

A crucial aspect for commenting on the pressure drop in different parts of the microvasculature is the correct labeling of vessel types. As mentioned, for a fully accurate distinction of vessel types the presence of smooth muscle cells and pericytes would have to be considered [25]. However, our labeling is solely based on morphology, topology and diameters and hence is error prone. A correct RBC trajectory flows through the different vessel types in the following order: PA → DA+A → C → V+AV → PV. To make the data analysis robust with respect to labeling errors we allow the RBCs to deviate from the correct path (PA → DA+A → C → V+AV → PV) for two subsequent branches, as long as it afterwards proceeds in the correct manner. In the course of this work we comment on the sensitivity of our results with respect to labeling errors (S3 Fig).

Only RBC trajectories which flow through the different vessel types in correct order: PA → DA+A → C → V+AV → PV are considered for data analysis. For analyses which only address flow phenomena in the capillary bed, (PA or DA+A) → C → (V+AV or PV) is also accepted as a correct RBC trajectory.

**Fig 2:**
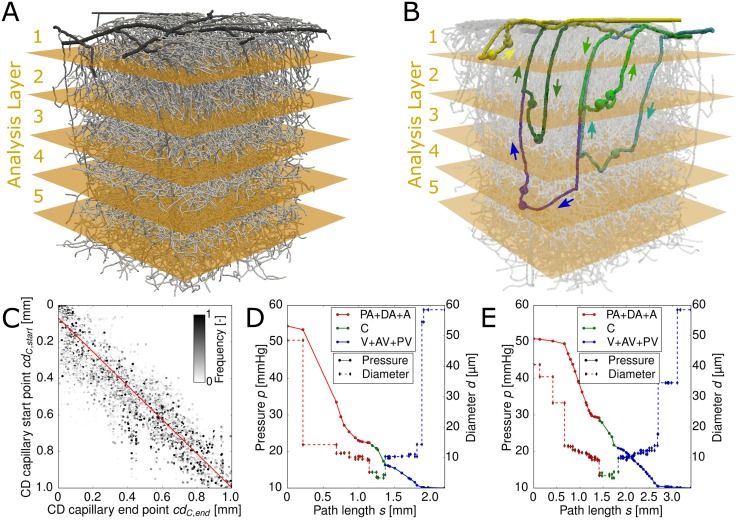
Pathway characteristics of red blood cells (RBCs) through cortical microvascular networks (MVNs). (A) MVN 1 with five 200 μm thick analysis layers (ALs). (B) Five exemplary RBC paths through MVN 1. The five paths enter the capillary bed in the 5 different ALs (yellow: AL 1, light green: AL 2, dark green: AL 3, light blue: AL 4, dark blue: AL 5). The spheres illustrate the start and the end points of the capillary bed along the underlying path. The flow direction in the DAs and the AVs are shown by the adjacent arrows. (C) Cortical depth of the capillary start point over the cortical depth of the capillary end point. The scatter plot shows all available end points for every start point. The color code represents the relative frequency of occurrence of the end point. The red line is a linear fit to the data points (underlying equation given in Eq (5)). The Pearson’s correlation coefficient is *r* = 0.86. (D) & (E) Pressure and diameter over the path length for two exemplary trajectories of two RBCs from MVN 1 with their capillary start point lying in AL 2 (light green RBC trajectory in (B)) and in AL 4 (light blue RBC trajectory in (B)), respectively. PA: pial artery, DA: descending arteriole, A: arteriole, C: capillary, V: venule, AV: ascending venule, PV: pial vein

In order to analyze the simulation results with respect to cortical depth we divided the MVN into five analysis layers (AL). We use 200 μm thick slices and limit our analysis to the upper 1000 μm of the cortex. MVN 1 with the five ALs is illustrated in Fig 2A.

Unless stated otherwise, all presented results have been averaged over all three MVNs analyzed.

## Results

Our analysis is based on trajectories of individual RBCs. On average 689 RBCs/ms enter the MVN at the pial arteries. Yet, only 21% of all tracked RBCs exhibit a full path (PA → DA+A → C → V+AV → PV). 70% of RBCs leave the network through an arteriole outlet (mainly at pial level) and 4% through a capillary outlet and can not be used for analysis purposes.

Flow phenomena in the capillary bed are the key focus of our investigations. The location where the RBC moves from an arteriole into the capillary bed is defined as the capillary start point. Capillary end points are the equivalent locations on the venule side, e.g. the location where the RBC leaves the capillary bed and enters a venule. The layer-specific analysis is based on the cortical depth of the capillary start point.

### Number of available pathways through the capillary bed varies over cortical depth

In a first step we analyzed the RBC pathways through the capillary bed. Fig 2B illustrates an exemplary RBC trajectory for each AL. Per MVN there are on average 2811 unique paths leading the RBCs from DA+A to V+AV. Yet, the distribution of the paths from DA+A to V+AV over the five ALs is not homogeneous (Table 4). The largest number of available paths is found for AL 2 and AL 3, followed by AL 1 and AL 4 where on average 580 unique paths exist between DA+A to V+AV. With approximately 200 available unique paths AL 5 offers the least possibilities for the RBCs to travel through the capillary bed. The nonhomogeneous distribution of paths through the capillary bed over cortical depth is the first evidence for layer-specific differences in the vasculature, which might as well affect functional properties.

**Table 4:** Number of available unique paths from DA+A to V+AV for the five ALs averaged over the RBC trajectories from 3 MVNs.

AL 1	AL 2	AL 3	AL 4	AL 5
558	862	862	601	201

The RBC trajectories are assigned to the different ALs based on the location of the capillary start point. DA: descending arteriole, A: arteriole, V: venule, AV: ascending venule, MVN: microvascular network, RBC: red blood cell, AL: analysis layer.

### Preferred pathways through the capillary bed

Per capillary start point there are on average 5 possible end points and 8 possible paths to reach the V+AV. To comment on the frequency a specific pathway is chosen, the preferred end point and the preferred path are introduced. If a capillary start point has *n*_*ep*_ end points the relative end point frequency *f*_*ep*_ is computed by dividing the number of RBCs reaching that end point by the total number of RBCs passing the capillary start point under investigation. A capillary start point has a preferred end point if the largest end point frequency is > 50% and the second largest one is < 30%. The equivalent definition is applied to define a preferred path. 60.7% of all capillary start points have a preferred end point and for 53.1% of all capillary start points there is a preferred path through the capillary bed. So even if the capillary bed is highly interconnected and offers a multitude of pathways through the capillary bed, the frequency at which different paths are chosen differs significantly. We assume that the nonhomogeneous perfusion in the baseline case enables the vasculature to more effectively alter perfusion during activation, e.g. by increasing the RBC flux through previously less perfused paths.

We next investigated whether the existence of the preferred end points emerges from topological characteristics of the MVN. We analyze if the relative frequencies of the available end points correlate with five different trajectory characteristics:

1. the Euclidean distances to the start point,
2. the average path lengths from the start to the end point,
3. the average sum of vessel resistances *R*_*ij*_ along the paths leading from the start to the end point,
4. the average flow rate along the paths and
5. the average RBC velocity along the paths.

For the trajectory characteristics (2)-(5) it has to be kept in mind that several paths might be leading from one start to one end point and hence averaging over all the available paths is necessary to obtain a single measure per end point. The trajectory characteristics have been normalized with the maximum value for each start point:

$$tc_{ep}^{norm}=\frac{tc_{ep}}{max(TC_{ep})},$$ (4)

where *tc*_*ep*_ is a trajectory characteristic of one path and *TC*_*ep*_ contains the trajectories characteristics for all end points of one start point. We compute Pearson’s correlation coefficients for the relative end point frequencies and the five normalized trajectory characteristics (Table 5).

**Table 5:** Pearson’s correlation coefficient for the relative end point frequencies and five trajectory characteristics averaged over 3 MVNs.

*r*_*distance*_	*r*_*path length*_	*r*_*resistance*_	*r*_*flow*_	*r*_*v*_*RBC*__
−0.01	−0.12	−0.23	0.44	0.50

Correlation coefficients for: euclidean distance: *r*_*distance*_, path length: *r*_*path length*_, sum of vessel resistances: *r*_*resistance*_, flow rate: *r*_*flow*_ and RBC velocity: *r*_*v*_*RBC*__.

The Euclidean distance is a pure topological measure and does not correlate with the relative end point frequencies. All further characteristics are based on the path of the RBCs through the capillary bed and hence not pure topological characteristics but influenced by the flow field. The average path length as well as the average sum of resistances along the path show a very weak negative correlation with the end point frequencies. However, with a correlation coefficient > 0.4 the relative end point frequencies correlate more strongly with the average flow rate and RBC velocity. This is plausible, because the average flow rate as well as the average RBC velocity have a direct impact on the distribution of the RBCs. More importantly, this result underlines the importance of a combined analysis of flow field and topology. A purely topological analysis might lead to wrong conclusion because it neglects crucial effects such as the RBC dynamics and the impact of the bifurcation rule.

### In-plane movement of RBCs in the capillary bed

In the next step we study the cortical depth *cd* of the capillary start points *cd*_*C*,*start*_ and the cortical depth of their capillary end points *cd*_*C*,*end*_. The Pearson’s correlation coefficient of *r* = 0.86 confirms that *cd*_*C*,*start*_ and the cortical depth of its capillary end points are strongly correlated. The linear fit for *cd*_*C*,*start*_ and *cd*_*C*,*end*_ reads as (red line in Fig 2C):

$$cd_{C,end}=1.08\;cd_{C,start}-0.08.$$ (5)

The y-intercept of −0.08 mm shows that the capillary end point is positioned closer to the cortical surface than its capillary start point. It is important to realize that the RBCs tend to move in-plane and no significant movement in the direction of the cortical depth takes place.

The in-plane movement of RBCs in the capillary bed is an important result because it implies that at the capillary level there is no significant flow between the different ALs and hence it justifies the approach to analyze flow characteristics with respect to different ALs. This is not only relevant for our simulations, but for all layer-specific analysis, including fMRI or bolus tracking measurements.

### One main feeding DA per AV

A further crucial aspect is the robustness of oxygen supply in the brain and the feeding regions of individual DAs. This topic has already been addressed in multiple numerical as well as experimental studies [42, 54, 55]. Although our sample size of available DAs and AVs is small we are able to make some observations about the feeding and draining regions of DAs and AVs, respectively.

In our study one DA feeds on average 3.8 different AVs and one AV is fed on average by 2.8 different DAs. Yet, one AV receives on average 72% of all its RBCs from one DA. This implies that every AV has a primary DA by which it is fed. Hence, the tissue around one AV might be very vulnerable to ischemia in case of occlusion of the primary feeding DA, unless significant blood flow reorganization takes place. This is in accordance with the observations of Nishimura et al. [56], which state that the “penetrating arterioles are a bottleneck in perfusion”.

### Pressure drop along RBC trajectories

A good understanding of the pressure distribution in MVNs is crucial to interpret the results of in vivo measurements and to derive mechanisms explaining the vasculature’s ability to up-regulate blood flow. Fig 2D and 2E depict the pressure along the path of two exemplary RBCs in MVN 1. The diameter along the RBC path is illustrated in line with the pressure, because the resistance of a vessel *R*_*ij*_ has a strong impact on the pressure drop and $R_{ij}\propto d_{ij}^{-4}$. In both examples the pressure drop becomes significant as soon as the vessel diameter falls below ∼ 15 μm. After an initial shoulder the slope of the pressure curve remains relatively constant until the RBC reaches the V+AV. The pressure drop tends to increase if the diameter of the vessel decreases. However, the diameter is not the only parameter influencing the pressure drop. Further very important quantities are the topology and connectivity of the vascular network as well as the number of RBCs in each vessel.

**Fig 3:**
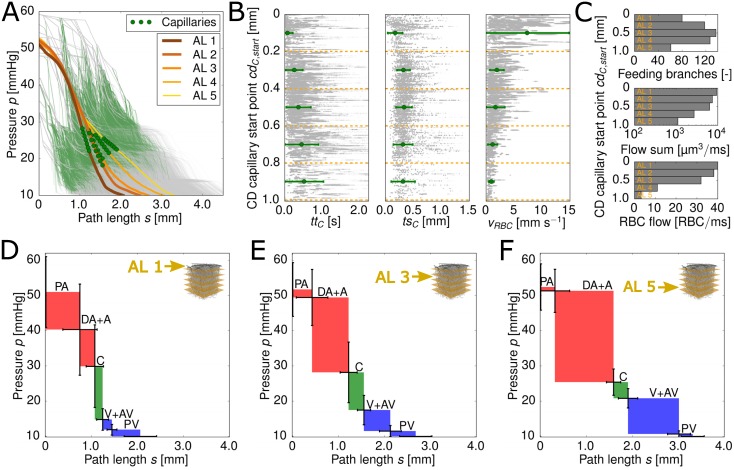
Pressure drop along the pathway of red blood cells (RBCs) and layer-specific flow characteristics. (A) Averaged pressure curves for the five analysis layers (ALs) (thick lines). The average locations of the capillaries are marked by green circles for each AL. The thin curves in the background represent the raw data and the thin green shading highlights the location of the capillaries for the raw data. (B) Capillary transit time *tt*_*C*_, transit path length *ts*_*C*_ and RBC velocity *v*_*RBC*_ for the five ALs (green symbols and error-bars, mean ± standard deviation). The scatter plot in the background shows the raw data for all MVNs studied. (C) Feeding properties for the five ALs. From top to bottom: (i) Number of feeding branches for the capillary bed, (ii) sum of blood flow rate through the feeding branches and (iii) sum of RBC flow rate through the feeding branches. (D),(E) & (F) Averaged pressure drop and averaged path length for different vessel types. The color coding facilitates the differentiation between arterioles (red), capillaries (green) and venules (blue). The standard deviation for the averaged pressure at the start of the different vessel types is given by the vertical error bars. The horizontal error bars represent the standard deviation for the averaged path length for the different vessel types. Fig (D),(E) and (F) show the averaged results for all RBCs with a capillary start point in AL 1, AL 3 and AL 5, respectively. PA: pial atery, DA: descending arteriole, A: arteriole, C: capillary, V: venule, AV: ascending venule, PV: pial vein

The pressure along the path of an RBC has been extracted for all RBCs with a correct pathway (PA → DA+A → C → V+AV → PV). To average the pressure curves the normalized path length is introduced:

$$s^{norm}=\frac{s}{s^{tot}},$$ (6)

where *s*^*tot*^ is the total length of the RBC path through the MVN. The RBCs are grouped based on the cortical depth of their capillary start point. An averaged pressure curve is computed for every AL (Fig 3A). In order to illustrate the total path length for the different ALs, the normalized path length is multiplied with the average total path length for each AL.

For all ALs the pressure starts to drop significantly after *s* ∼ 0.5 mm. As the capillary bed starts on average at *s* ∼ 1.3 mm (depending on the AL), the pressure already starts to drop in the arterioles. The shape of the averaged pressure curves for AL 1–3 (*cd* < 0.6 mm) is very similar. All three curves have an S-shape with a constant slope in the middle of the path and a flatter slope for the first and the last 0.5 mm along the path. Even for AL 4–5 (*cd* ≥ 0.6 mm) the shapes of the curves are comparable to the one of AL 1–3. However, the slope flattens shortly before the start of the capillary bed (*s* ∼ 1.2 mm). This is not seen for the pressure curves of AL 1–3. The difference between the total path length in AL 1 and AL 5 is 1.25 mm.

It might seem surprising that the pressure drops continuously and no steeper pressure drop in the capillaries is observed, even though that is where the smallest vessel diameters are found. We assume that this can partly be attributed to averaging over > 1 million RBC pathways, which leads to a smoothing of the originally bumpy curves (Fig 2D and 2E for comparison). The averaged diameter along the path of the RBCs shows a continuous drop in diameter until the capillary bed is reached (S4 Fig). It is smallest at the beginning of the capillary bed and starts to increase for downstream vessels for all ALs. Surprisingly, the pressure drops continuously along the whole path even though the average diameter exhibits strong variations over the path (S4 Fig). This confirms that the pressure drop is not only affected by the diameter, but as aforementioned by the connectivity of the MVN and the distribution of RBCs in the MVN.

### Layer-specific location of largest pressure drop

The location of the largest pressure drop is the ideal region for regulating hemodynamics [3]. Hence, to comment in more detail on the contribution of different vessel types to the total pressure drop the pressure drop per vessel type is computed (Fig 3D–3F). We average the pressure at the inlet vertices of the five different vessel types (PA, DA+A, C, V+AV, PV). Additionally, the averaged path length is computed for each vessel type. As for the previous studies, we calculate averages for every AL based on the cortical depth of the capillary start point. As the sample size of PAs and PVs is very small, the pressure drops are only illustrated for completeness and are not analyzed further.

The differences between the three ALs illustrated are striking. Whereas in AL 1 (*cd* = 0 − 0.2 mm) the pressure drop in the capillaries is dominant (37%), in AL 3 (*cd* = 0.4 − 0.6 mm) and AL 5 (*cd* = 0.8 − 1.0 mm) the largest pressure drop is found in the DA+A (AL 3: 51% and AL 5: 61%). This reveals that the contribution of the different vessel types to the total pressure drop is highly dependent on the cortical depth of the capillary start point. Furthermore, our results show that total pressure drop in the DA+A correlates with the path length traveled in the DA+A. The total path length increases the deeper the RBC flows into the MVN (Fig 3D–3F).

To address the sensitivity to labeling errors we compare the results of the standard labeling to two modified labelings (S3 Fig). For deep ALs the result is very robust (S3D–S3F Fig). A larger sensitivity is found for the labeling of capillaries and arterioles in AL 1 (S3A–S3C Fig). For all investigated scenarios the capillaries play a significant role for the pressure drop in AL 1. However, if S3A and S3B Fig are compared, it becomes evident that on average 31% of the total pressure drop in the capillary bed takes place in the first capillary branch. Hence, for AL 1 where the pressure gradients are very steep a correct labeling is crucial.

Many studies related to neurovascular coupling differentiate between the main branch of the DA and the A branching off (sometimes called precapillary arterioles) [57, 58]. Our results show that for AL 2–5 the pressure drop mainly takes place in the DAs and is very small in the As (S1 Table). For AL 1 the pressure drop in the DAs and As is comparable. Based on the original labeling 16% of all RBCs flow directly from the DAs into the capillary bed. We conclude that already in the main branch of the DAs the pressure starts to drop significantly.

All in all, our results show that it is indispensable to account for the pressure drop in the DAs+As. Generalizations on the location of the largest pressure drop in the vasculature are not valid because it needs to be differed based on the cortical depth of the capillary start point. For AL 1–2 the location of the largest pressure drop is the capillary bed and to be more precise mainly the first branches of the capillary bed. For AL 3–5 the pressure drops the most in the DAs+As. The pressure drop in the Cs decreases with increasing cortical depth.

### Layer-specific flow characteristics affecting oxygen discharge

Oxygen discharge from the vasculature to tissue is a diffusion driven process which based on common notion mainly takes place in the capillary bed. However, this view has been recently challenged by Sakadžić et al. [58] who state that 50% of the oxygen is extracted from arterioles in the baseline case. Nonetheless, the impact of the heterogeneous flow properties in the capillary bed are crucial to fully understand the oxygen supply of the brain. For example Rasmussen et al. [59] showed that a reduced CTH leads to an increase in OEF.

Additionally, our presented results show that significant differences between the ALs persist and hence, the question arises if these differences also affect flow phenomena observed in the capillary bed. We present results for capillary transit time *tt*_*C*_, capillary transit path length *ts*_*C*_ and the capillary RBC velocity *v*_*RBC*,*C*_ in different ALs (Fig 3B). All three quantities have an impact on the amount of oxygen which can be discharged from individual RBCs.

The mean transit path in the capillary bed is shortest for AL 1 (∼0.17 mm). For all other ALs it is approximately constant *ts*_*c*_ ≈ 0.3 mm. This agrees well with the observations of Sakadžić et al. [58] which find an average path length of 0.34 mm for measurements until a depth of 0.45 mm. To the best of our knowledge Kleinfeld et al. [11] and Gutiérrez-Jiménez et al. [14] performed the only measurements of RBC velocity up to a depth of 600 μm [11, 14]. In both works a slightly decreased RBC velocity over the cortical depth is observed. This agrees well with our results illustrated in Fig 3B. The changes in capillary RBC velocity over depth probably result from the high pressure gradient between DA+A and V+AV close to the surface, which decreases with cortical depth. For AL 1 we obtain an average RBC velocity which is larger than the velocity range stated in literature (0.4 − 1.0 mm s^−1^ [4, 10, 11, 13, 51]). The capillaries with the large RBC velocities are located very close to the cortical surface (*cd*_*C*,*start*_ < 75 μm) and connect DAs with nearby AVs, hence the capillary transit path length is comparably short.

The mean capillary transit time on the other hand increases with cortical depth, which is in accordance with the higher capillary RBC velocity *v*_*RBC*,*C*_ close to the cortical surface. For AL 1 the mean capillary transit time is equal to 0.07 ± 0.14 s and rises to 0.52 ± 0.51 s for AL 5. Literature values for capillary transit time are sparse. The most recent and suitable measurements have been done by Gutiérrez-Jiménez et al. [14] which obtain a mean transit time of 0.66 ± 0.20 s from arterioles to venules.

The small capillary transit times close to the cortical surface suggest that the RBCs have less time to discharge oxygen and hence the OEF per RBC is smaller at the cortical surface than deep in the cortex. As the average transit path length *ts*_*C*_ is nearly constant over depth, the increased transit time *tt*_*C*_ with depth is also reflected in a decrease in RBC velocity *v*_*RBC*_ with depth (Fig 3B). The RBC velocity is proportional to the RBC flow rate, which is confirmed by Fig 3C. All in all, our results suggest that the oxygen level in the tissue is largest close to the cortical surface.

Due to the large RBC feeding flow in AL 1 and the large RBC velocities, it seems likely that RBCs traveling close to the cortical surface are still highly saturated with oxygen as they reach the venules. As the feeding flow rate decreases for deeper ALs (Fig 3C) and the transit time increases, we expect the RBCs leaving the capillary bed at a deeper cortical level to be less saturated with oxygen than the RBCs close to the cortical surface.

The scatter plot in Fig 3B confirms the presence of a large CTH in cerebral MVNs. While for the capillary transit path the standard deviation remains constant over cortical depth, it increases for the capillary transit time. Consequently, deeper in the cortex the reduction of CTH might be a more effective mean to increase the OEF than close to the cortical surface. Furthermore, the standard deviation of the capillary transit time and the RBC velocity show opposing trends over cortical depth. Those differences have to be kept in mind for estimations of CTH based on the RBC velocity.

In Fig 3C the total number of feeding branches for the five ALs, the sum of the blood flow rate and the sum of the RBC flow rate through those branches is illustrated. While the blood flow and the RBC flow rate show similar trends, namely a lower feeding flow rate for deeper ALs, the number of feeding branches increases until AL 3 and then drops significantly. The number of feeding branches is a topologically based quantity, while the feeding flow rates are flow related quantities. Both results underline once more the complex flow phenomena in MVNs and the necessity to consider the flow field and the vascular topology as one entity.

## Discussion

We simulated blood flow in three realistic MVNs from the mouse parietal cerebral cortex. To our knowledge this is the first numerical work in realistic MVNs where RBCs are tracked individually and the results are directly based on RBC trajectories. As the RBCs are the brain’s oxygen source they are the most fundamental entity of blood for the up-regulation of oxygen and hence should be at the basis of a functional analysis. The focus of our investigations lies on the pressure field in the MVN and on depth specific flow characteristics.

Our results show that the location of the maximal pressure drop depends significantly on the cortical depth of the capillary start point. While close to the cortical surface the pressure drop in the capillary bed is dominant, the deeper we dive into the cortex the larger the pressure drop in the DAs+As. This is plausible because based on the vascular topology the distance a RBC travels in the DAs increases for deeper layers and hence a larger pressure drop in DAs+As is to be expected.

In Guibert et al.’s [60] numerical work the pressure drop in different vessel generations is studied in the primate cortex. They show that the pressure drops rapidly over the first vessel generations and then reaches a fairly constant level. Unfortunately, no detailed differentiation between vessel types is presented and differences over depth are neglected, such that a quantitative comparison with our results is difficult. Qualitatively our results agree well with the steep pressure drop early along the path of the RBC. However, we do not observe the pressure plateau in the capillary bed as stated by Guibert et al. [60] but a continuous drop in pressure until the Vs+AVs are reached (Fig 3A). We suppose that the difference results from grouping the capillaries into generations instead of analyzing the pressure drop over the RBC path. Furthermore, a species difference between the primate and rodent cannot be ruled out, which makes a direct comparison even more difficult.

Blinder et al. [19] computed the average resistance of the microvasculature in cortical layer IV as well as the average resistance for DAs and AVs from the surface to cortical layer IV. They obtained an average resistance of 0.1, 0.4 and 0.2P μm^3^ for the DAs, the capillary bed and the AVs, respectively. If pure plasma flow is assumed, the largest pressure drop would take place in the capillary bed [3]. This disagrees with our observations for AL 3 (≙ cortical layer IV), where the pressure drop is largest for the DAs+As (51%) (Fig 3E). We assume that those discrepancies result from the fully topological analysis in [19]. For computing the average resistance of the capillary bed 1000 randomly chosen pairs of vertices were used. We suggest that it is more appropriate to base the choice of vertices on actual RBC paths, because topologically connected but not functionally connected vertex pairs might artificially increase the average resistance of the capillary bed.

In a recent numerical study by Gould et al. [43] the pressure drop was analyzed along possible paths through the cortical microvasculature. While the focus was on predicting oxygen levels in the brain, one of their main claim is that the “capillary bed offers the largest hemodynamic resistance to the cortical blood supply”. This is partly in contrast to our observations, especially for deeper ALs. Gould et al. applied no flow boundary conditions for the deep in- and outflows that, based on [42], can lead to an underestimation of flow and thus can impact the resulting pressure field. A more quantitative comparison is however difficult, because values for the pressure drop per vessel type are not provided. Furthermore, whereas two of the analysed networks support their conclusion, it is less obvious for the remaining two. We believe that analyzing the pressure drop over the path length of discrete RBC trajectories is more robust and that a layer specific analysis provides further important information.

Consequently, we challenge the hypothesis that the dilation of individual capillaries leads to a flow increase of 84% and therewith would be the ideal location for vascular changes during activation [3]. Based on our results in AL 3–5 the DA+A is the location with the largest pressure drop and hence the most crucial for up-regulating flow. For AL 1–2 the situation is slightly different and vascular changes in the capillary bed and the DA+A could both be effective means to increase the flow rate. However, the number of capillaries is significantly larger than the number of arterioles and hence a well coordinated response of multiple capillaries could compensate for the smaller pressure drop. The impact of the possible regulation scenarios has to be analyzed in future numerical studies.

We postulate that different regulation mechanisms are playing at different cortical depths and with different objectives. The dilation of DAs+As seems to be the most relevant mechanisms to increase the flow rate on a large scale, whereas capillary dilation might play a more crucial role for small scale and very localized regulations such as redistribution of RBCs or flow homogenization.

On the larger scale Zhao et al. [21] and Goense et al. [20] showed that layer-specific regulation mechanisms have to exist in order to explain the laminar differences in CBV and CBF change during activation. Gutiérrez-Jiménez et al. [14] addressed this topic more locally by measuring RBC velocity and flux in capillaries until a cortical depth of 450 μm during baseline and electrical forepaw stimulation. They show that the RBC velocity and RBC flux do not change homogeneously over depth during activation. This agrees well with our hypothesis that different regulation mechanisms are playing at different depths and that the microvasculature plays a significant role in neurovascular coupling.

The question arises whether the layer-specific differences in the pressure drop can be explained by topological characteristics. We address this issue by analyzing differences in RBC pathways, in capillary transit time and transit path lengths and in the feeding of the different layers.

Our results show that for AL 2–3 the RBCs have the most options to travel through the capillary bed. These ALs (200 − 600 μm) which overlap with the granular layer IV in the mouse somatosensory cortex also show the largest number of feeding branches (Fig 3C). Furthermore, our findings are in accordance with results obtained in the macaque visual cortex [16], where the cortical layer IV (and in particular IVcB) shows the highest vascular density. A more interconnected microvasculature in AL 2–3 would be beneficial to up-regulate the oxygen supply with a high spatial precision. It seems plausible that the brain has an improved ability to locally up-regulate oxygen in layer IV in the granular cortex, which has the highest neuronal density and metabolic demand.

Due to the high neuronal density in cortical layer IV one might assume that the feeding flow rate in this area is largest. Yet, we observe a decrease in feeding blood and RBC flow over cortical depth. While close to the surface the RBC flow is very large and the RBCs as well as the tissue are most likely highly saturated with oxygen, deeper in the cortex a more efficient extraction seems to be crucial to avoid hypoxic tissue regions. This implies that deeper cortical layers might be more vulnerable to disruptions in feeding flow, because the safety margin in RBC flow seems to be significantly lower than close to the cortical surface.

Various studies measured the tissue and plasma oxygenation over cortical depth. However, results differ significantly. While Lyons et al. [61] observe an increase in tissue oxygen partial pressure (PO_2_) until cortical layer IV, Sakadžić et al. [58] and Devor et al. [58, 61, 62] report the highest PO_2_ in cortical layer I. The reason for the discrepancies are not yet resolved, but Lyons et al. suggest it might result from anesthesia effects. A further topological explanation for the results obtained in [61] could be a very coarse capillary grid in AL 1 which impedes the diffusion of oxygen.

Based on our simulation results with a large feeding flow rate in AL 1 and comparably short transit times, a high tissue PO_2_ in AL 1 seems more plausible. However, our hypothesis is solely based on the results of blood flow simulations, which do not account for oxygen discharge from capillaries. Further measurements and simulations with oxygen discharge from capillaries are necessary to properly answer this question.

We postulate that RBCs leaving the capillary bed deep in the cortex will be less saturated with oxygen than RBCs close to the surface. This is in line with the observations in [58].

Furthermore, the large feeding flow rates for the ALs close to the cortical surface suggest that up-regulation of flow might be less critical close to the cortical surface, because the level of highly-oxygenated blood supplied is very large and the neuronal energy demand is comparably low and constant. We hypothesize that up-regulation of flow is mainly relevant for deeper ALs to match the metabolic demand of the corresponding neuronal layer. This seems to be in line with the work of Tian et al. [6] who observes that the delay in vascular response to stimulation is smallest for deeper cortical layers.

As previously mentioned, in the sensory cortex AL 2–3 overlap with cortical layer IV which has a high neuronal density and a higher and more fluctuating energy demand. One might speculate that the vasculature is designed to primarily support hemodynamic regulation at that depth.

Another means to improve the oxygenation of the tissue is an increased OEF. As mentioned above homogenization in RBC flow is beneficial for the oxygen extraction [59]. However, during baseline it is well known that the flow in the microvasculature is very heterogeneous [4, 10–13], which is confirmed by our results. We postulate that flow homogenization is more relevant at deeper cortical levels, where the feeding flow rate is lower and the CTH is larger. This agrees with the decreased coefficient of variation of capillary transit times observed for *cd* > 200 μm due to activation [14]. Consequently, our results support the hypothesis that flow homogenization could be an effective mean to up-regulate the oxygen supply. Even if experimental studies on flow homogenization are currently mainly executed for the upper part of the cortex, we postulate that flow homogenization might be even more effective at deeper cortical levels, where the overall saturation of RBCs is expected to be lower, and hence a very effective discharge of oxygen is required.

Based on our results several conclusions can be drawn: (1) The location of the largest pressure drop is a function of cortical depth and hence its impact on CBF increase is also depth dependent. (2) Laminar differences exist for all relevant flow characteristics, such as capillary transit time, feeding flow and RBC velocity. (3) In order to improve the understanding of the flow in MVNs it is crucial to consider the vascular topology and the flow and pressure distribution as one entity. Purely topological analysis might result in spurious interpretations and hence should be avoided.

Further experimental and numerical studies will be needed to further investigate layer-specific effects on hemodynamic regulation, because averaging over the whole network might wash out layer-specific effects which are crucial to properly understand neurovascular coupling.

## Supporting information

S1 TextEmpirical relation for the computation of the relative apparent viscosity as stated in Pries et al. [34].All available in vivo formulations for the computation of the relative apparent viscosity are based on networks from the mesentery and have been optimized for a diameter range of 4 − 40 μm. However, the topology of the cerebral microvasculature is completely different and 33% of the vessel diameters in the three MVNs analyzed are < 4.0 μm. For that reason we consider the in vitro formulation as the most suitable choice to account for the presence of RBCs.For an improved readability the edge indices *ij* are not shown in the following equations.
$$\mu_{vitro}=1+\left(\mu_{0.45}-1\right)\;\frac{{\left(1-hd\right)}^{C}-1}{{\left(1-0.45\right)}^{C}-1}$$(7)
with
$$\mu_{0.45}=220\;e^{-1.3\;d}+3.2-2.44\;e^{-0.06\;d^{0.645}}$$(8)
and
$$C=\left(0.8+e^{-0.075\;d}\right)\cdot \left(-1+\frac{1}{1+10^{-11}\;d^{12}}\right)+\frac{1}{1+10^{-11}\;d^{12}}$$(9)
The discharge hematocrit *hd* is computed from the tube hematocrit *ht* ([23]):
$\frac{ht}{hd}=hd+\left(1-hd\right)\;\left(1+1.7\;e^{-0.415\;d}-0.6\;e^{-0.011\;d}\right)$(10)(PDF)

S1 FigHistogram-based upscaling approach of capillary vessel diameters based on a beta distribution.The schematical drawing illustrates key steps and variables of the histrogram-based upscaling approach for one bin *i*. It is implemented as follows:(1) The diameter range of the beta distribution [2.5 μm, 9.0 μm] is divided into 500 bins (bin width = 0.013 μm). The lower and upper bound of bin *i* are denoted $d_{i}^{min}$ and $d_{i}^{max}$ (panel A).(2) The upscaling approach starts with bin *i* = 0 containing the smallest vessel diameters and step by step proceeds to larger vessel diameters.(3) The desired number of vessels in bin *i* ($N_{i}^{goal,\beta }$) is computed based on the beta distribution (panel A).(4) Based on the current diameter bound the number of vessels in bin *i* $N_{i}^{current}$ is computed (panel A).(4.1) If $N_{i}^{goal,\beta }\geq N_{i}^{current}$ we proceed to the next bin.(4.2) If $N_{i}^{current}>N_{i}^{goal,\beta }$ the diameters need to be upscaled. Therefor, all vessels in $N_{i}^{current}$ are sorted by their diameter (panel B). $d_{i}^{goal,cut}$ is the diameter for which we obtain $N_{i}^{current}=N_{i}^{goal,\beta }$ (panel B). All vessels with a diameter $>d_{i}^{goal,cut}$ are upscaled by adding the constant $\Delta d_{i}^{upscale}$. $\Delta d_{i}^{upscale}$ is the difference between $d_{i}^{max}$ and $d_{i}^{goal,cut}$. We obtain $N_{i}^{goal,\beta }=N_{i}^{current}$ and proceed to bin *i* + 1.(EPS)

S2 FigImpact of RBCs on the flow rate in capillaries and non-capillaries in MVN 1.We compared the flow in networks with the same boundary conditions but for pure plasma flow without RBCs. To measure the impact of RBCs the flow ratio for every vessel is computed. The flow ratio *γ*_*ij*_ is defined as $\gamma_{ij}=\frac{q_{ij}^{plasma}}{\mu_{vitro,ij}\;q_{ij}^{blood}}$. If the presence of RBCs would not interact with the distribution of flow rates and would only lead to a homogeneously increased resistance the flow ratio would be equal to 1 in every vessel. Panel A depicts the distribution of the flow ratio in capillaries, Panel B shows the same for non-capillaries. In both panels, the darker shaded bars represents vessels where $q_{ij}^{plasma}$ and $\mu_{vitro,ij}\;q_{ij}^{blood}$ differ less than 5%. We find only 17.8% of the capillaries and 29.0% for the larger vessels where this is the case. The median for the relative difference between $q_{ij}^{plasma}$ and $\mu_{vitro,ij}\;q_{ij}^{blood}$ is 15.0% in capillaries and 9.4% in non-capillaries. These results confirm a significant effect of RBCs on the flow. This effect is more prominent in capillaries than in larger vessels.(EPS)

S3 Fig Sensitivity analysis for the labeling of the vessel types based on the layer-specific average pressure drop per vessel type. The results of the standard labeling (A and D) are compared to two modified labeling approaches (B and E, C and F). As the largest pressure drop takes place either in the DAs+As or the Cs we are mainly interested in a correct differentiation between DA+A and C. Hence, our sensitivity analysis focuses on the region where the vessel type changes from DA+A to C. In the first modified labeling the first C along the RBC paths is considered as a DA+A (B and E). For the second modified labeling the last DA+A branch is assigned to the C (C and F). We compare the results for the analysis layer (AL) at the cortical surface and the AL deepest in the cortex (AL 1 and AL 5). PA: pial atery, DA: descending arteriole, A: arteriole, C: capillary, V: venule, AV: ascending venule, PV: pial vein
(EPS)

S4 Fig Averaged vessel diameter over the red blood cell paths for the five analysis layers (ALs). Averaged diameter curves for the five ALs. The average locations of the capillaries are marked by green circles for each AL. The averaging procedure is similar to that described for Fig 3A.
(EPS)

S1 Table Pressure drop in descending arterioles (DAs) and arterioles (As) for the five analysis layers (ALs). We differentiate between DA and A by tracking the DA from its starting point and applying an angle criterion. As soon as the angle between two subsequent branches is smaller than 125° all following vessels are considered as A. The pressure drop was averaged similar to the results presented in Fig 3D–3F.
(PDF)
