# Supplementary material for: Depth-dependent flow and pressure characteristics in cortical microvascular networks
Source: PLoS Comput Biol. 2017 Feb 14;13(2):e1005392. doi: 10.1371/journal.pcbi.1005392 (PMC5347440; doi:10.1371/journal.pcbi.1005392)
Supplement: S1 Text — All available in vivo formulations for the computation of the relative apparent viscosity are based on networks from the mesentery and have been optimized for a diameter range of 4 − 40 μm. However, the topology of the cerebral microvasculature is completely different and 33% of the vessel diameters in the three MVNs analyzed are < 4.0 μm. For that reason we consider the in vitro formulation as the most suitable choice to account for the presence of RBCs. For an improved readability the edge indices ij are not shown in the following equations. μvitro=1+(μ0.45-1)(1-hd)C-1(1-0.45)C-1(7) with μ0.45=220e-1.3d+3.2-2.44e-0.06d0.645(8) and C=(0.8+e-0.075d)·(-1+11+10-11d12)+11+10-11d12(9) The discharge hematocrit hd is computed from the tube hematocrit ht ([23]): hthd=hd+(1-hd)(1+1.7e-0.415d-0.6e-0.011d)(10) (PDF) [file pcbi.1005392.s001.pdf]

Supporting Information

S1 Text

S1 Text. Empirical relation for the computation of the relative apparent viscosity as stated in Pries et al. [34]. All available in vivo formulations for the computation of the relative apparent viscosity are based on networks from the mesentery and have been optimized for a diameter range of $4 - 40\,\mu m$. However, the topology of the cerebral microvasculature is completely different and 33% of the vessel diameters in the three MVNs analyzed are $< 4.0\,\mu m$. For that reason we consider the in vitro formulation as the most suitable choice to account for the presence of RBCs.

For an improved readability the edge indices ij are not shown in the following equations.

$$\mu_{vitro} = 1 + (\mu_{0.45} - 1)\,\frac{(1-hd)^C - 1}{(1-0.45)^C - 1} \tag{1}$$

with

$$\mu_{0.45} = 220\,e^{-1.3\,d} + 3.2 - 2.44\,e^{-0.06\,d^{0.645}} \tag{2}$$

and

$$C = (0.8 + e^{-0.075\,d}) \cdot (-1 + \frac{1}{1 + 10^{-11}\,d^{12}}) + \frac{1}{1 + 10^{-11}\,d^{12}} \tag{3}$$

The discharge hematocrit hd is computed from the tube hematocrit ht ([?]):

$$\frac{ht}{hd} = hd + (1-hd)\,(1 + 1.7\,e^{-0.415\,d} - 0.6\,e^{-0.011\,d}) \tag{4}$$

S1 Fig

Histogram-based upscaling approach of capillary vessel diameters based on a beta distribution.

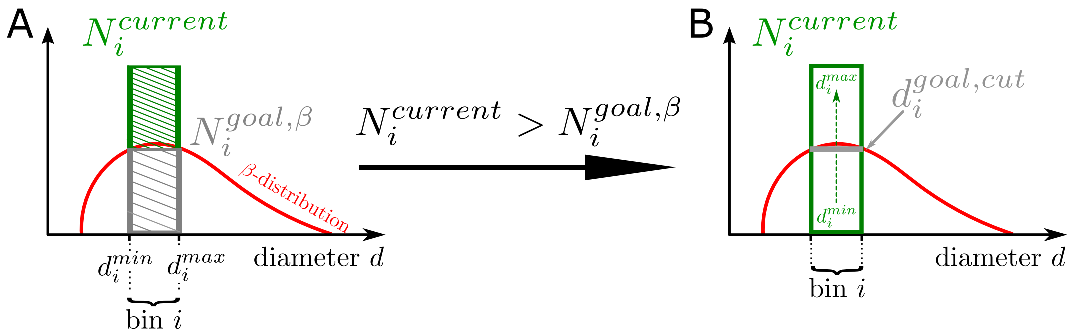

S1 Fig. The schematical drawing illustrates key steps and variables of the histrogram-based upscaling approach for one bin i. It is implemented as follows:
(1) The diameter range of the beta distribution $[2.5\,\mu m, 9.0\,\mu m]$ is divided into 500 bins (bin width = $0.013\,\mu m$). The lower and upper bound of bin i are denoted d_i^{min} and d_i^{max} (panel A).
(2) The upscaling approach starts with bin $i = 0$ containing the smallest vessel diameters and step by step proceeds to larger vessel diameters.
(3) The desired number of vessels in bin i ($N_i^{goal,\beta}$) is computed based on the beta distribution (panel A).
(4) Based on the current diameter bound the number of vessels in bin i $N_i^{current}$ is computed (panel A).
(4.1) If $N_i^{goal,\beta} \geq N_i^{current}$ we proceed to the next bin.
(4.2) If $N_i^{current} > N_i^{goal,\beta}$ the diameters need to be upscaled. Therefor, all vessels in $N_i^{current}$ are sorted by their diameter (panel B). $d_i^{goal,cut}$ is the diameter for which we obtain $N_i^{current} = N_i^{goal,\beta}$ (panel B). All vessels with a diameter $> d_i^{goal,cut}$ are upscaled by adding the constant $\Delta d_i^{upscale}$. $\Delta d_i^{upscale}$ is the difference between d_i^{max} and $d_i^{goal,cut}$. We obtain $N_i^{goal,\beta} = N_i^{current}$ and proceed to bin $i + 1$.

S2 Fig

Impact of RBCs on the flow rate in capillaries and non-capillaries in MVN 1.

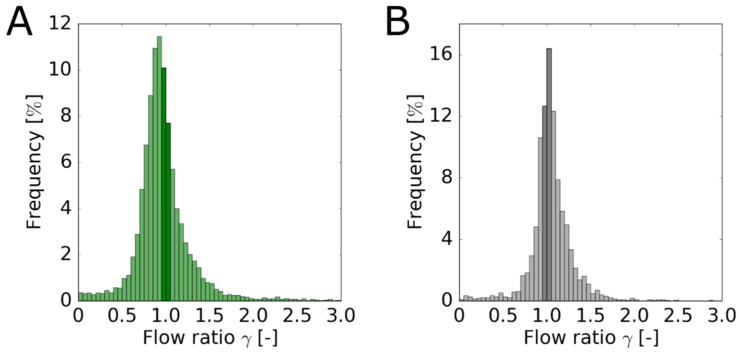

S2 Fig. We compared the flow in networks with the same boundary conditions but for pure plasma flow without RBCs. To measure the impact of RBCs the flow ratio for every vessel is computed. The flow ratio γ_{ij} is defined as $\gamma_{ij} = \frac{q_{ij}^{plasma}}{\mu_{vitro,ij}\, q_{ij}^{blood}}$. If the presence of RBCs would not interact with the distribution of flow rates and would only lead to a homogeneously increased resistance the flow ratio would be equal to 1 in every vessel. Panel A depicts the distribution of the flow ratio in capillaries, Panel B shows the same for non-capillaries. In both panels, the darker shaded bars represents vessels where q_{ij}^{plasma} and $\mu_{vitro,ij}\, q_{ij}^{blood}$ differ less than 5 %. We find only 17.8 % of the capillaries and 29.0 % for the larger vessels where this is the case. The median for the relative difference between q_{ij}^{plasma} and $\mu_{vitro,ij}\, q_{ij}^{blood}$ is 15.0 % in capillaries and 9.4 % in non-capillaries. These results confirm a significant effect of RBCs on the flow. This effect is more prominent in capillaries than in larger vessels.

S3 Fig

Sensitivity analysis for the labeling of the vessel types based on the layer-specific average pressure drop per vessel type.

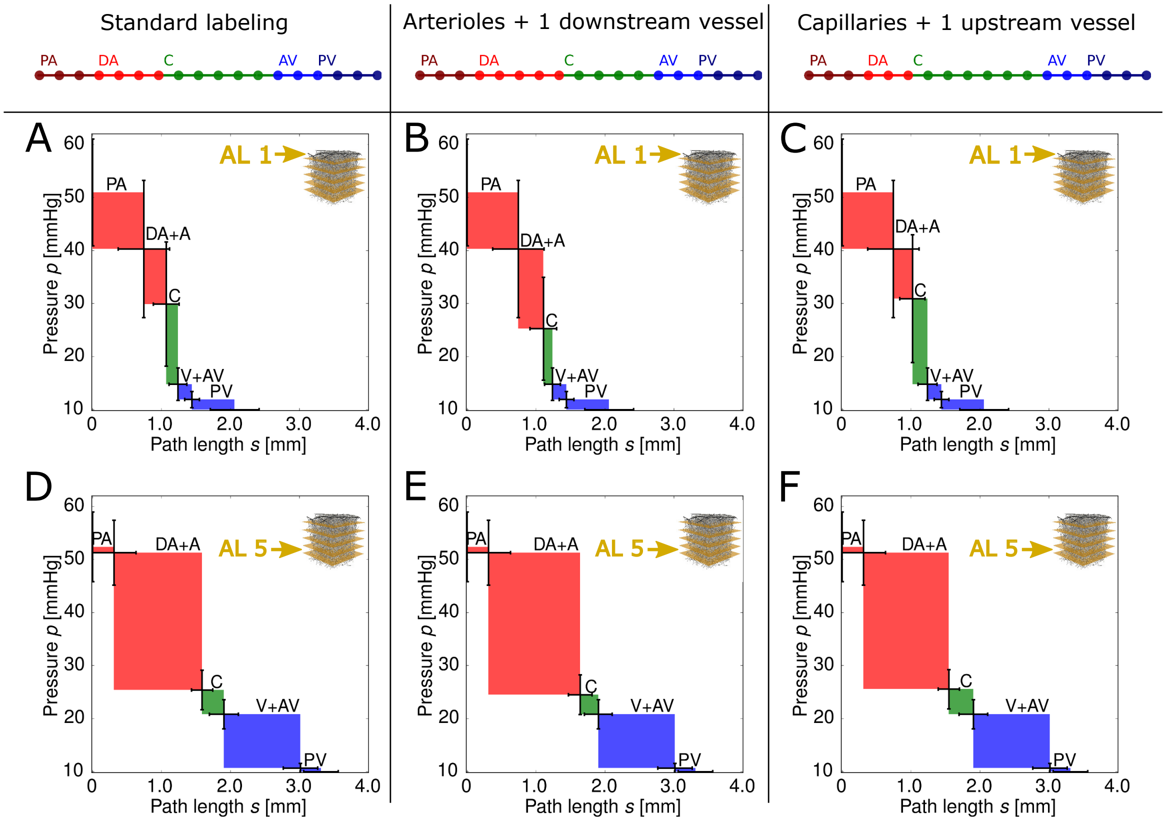

S3 Fig. The results of the standard labeling (A and D) are compared to two modified labeling approaches (B and E, C and F). As the largest pressure drop takes place either in the DAs+As or the Cs we are mainly interested in a correct differentiation between DA+A and C. Hence, our sensitivity analysis focuses on the region where the vessel type changes from DA+A to C. In the first modified labeling the first C along the RBC paths is considered as a DA+A (B and E). For the second modified labeling the last DA+A branch is assigned to the C (C and F). We compare the results for the analysis layer (AL) at the cortical surface and the AL deepest in the cortex (AL 1 and AL 5). PA: pial atery, DA: descending arteriole, A: arteriole, C: capillary, V: venule, AV: ascending venule, PV: pial vein

S4 Fig

Averaged vessel diameter over the red blood cell paths for the five analysis layers (ALs).

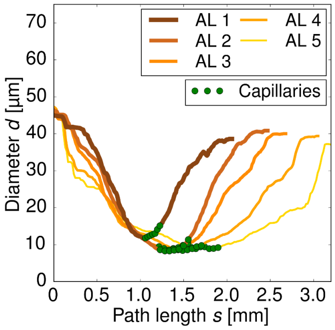

S4 Fig. Averaged diameter curves for the five ALs. The average locations of the capillaries are marked by green circles for each AL. The averaging procedure is similar to that described for Fig **??**A.

S1 Table

S1 Table. Pressure drop in descending arterioles (DAs) and arterioles (As) for the five analysis layers (ALs). We differentiate between DA and A by tracking the DA from its starting point and applying an angle criterion. As soon as the angle between two subsequent branches is smaller than 125° all following vessels are considered as A. The pressure drop was averaged similar to the results presented in Fig **??** D-F.

**Vessel type**	**AL 1**	**AL 2**	**AL 3**	**AL 4**	**AL 5**
DA	5.0 mmHg	16.2 mmHg	18.8 mmHg	21.4 mmHg	24.3 mmHg
A	6.5 mmHg	5.2 mmHg	3.0 mmHg	1.9 mmHg	1.8 mmHg
