# Supplementary material for: Depth-dependent flow and pressure characteristics in cortical microvascular networks
Source: PLoS Comput Biol. 2017 Feb 14;13(2):e1005392. doi: 10.1371/journal.pcbi.1005392 (PMC5347440; doi:10.1371/journal.pcbi.1005392)
Supplement: S1 Table — We differentiate between DA and A by tracking the DA from its starting point and applying an angle criterion. As soon as the angle between two subsequent branches is smaller than 125° all following vessels are considered as A. The pressure drop was averaged similar to the results presented in Fig 3D–3F. (PDF) [file pcbi.1005392.s006.pdf]

S1 Table

S1 Table. Pressure drop in descending arterioles (DAs) and arterioles (As) for the five analysis layers (ALs). We differentiate between DA and A by tracking the DA from its starting point and applying an angle criterion. As soon as the angle between two subsequent branches is smaller than 125° all following vessels are considered as A. The pressure drop was averaged similar to the results presented in Fig 3 D-F.

Vessel type	AL 1	AL 2	AL 3	AL 4	AL 5
DA	5.0 mmHg	16.2 mmHg	18.8 mmHg	21.4 mmHg	24.3 mmHg
A	6.5 mmHg	5.2 mmHg	3.0 mmHg	1.9 mmHg	1.8 mmHg
